# Measles transmission during a large outbreak in California

**DOI:** 10.1016/j.epidem.2019.100375

**Published:** 2019-11-10

**Authors:** Lee Worden, Sarah F. Ackley, Jennifer Zipprich, Kathleen Harriman, Wayne T.A. Enanoria, Rae Wannier, Travis C. Porco

**Affiliations:** a Francis I. Proctor Foundation, University of California, San Francisco, CA, USA; b Dept. of Epidemiology and Biostatistics, University of California, San Francisco, CA, USA; c California Dept. of Public Health, Immunization Branch, 850 Marina Bay Parkway, Building P, Richmond, CA, USA

**Keywords:** Vaccination, Immunization, MMR, Subexponential transmission, Wallinga-Teunis estimation

## Abstract

A large measles outbreak in 2014–2015, linked to Disneyland theme parks, attracted international attention, and led to changes in California vaccine policy. We use dates of symptom onset and known epidemic links for California cases in this outbreak to estimate time-varying transmission in the outbreak, and to estimate generation membership of cases probabilistically. We find that transmission declined significantly during the course of the outbreak (*p* = 0.012), despite also finding that estimates of transmission rate by day or by generation can overestimate temporal decline. We additionally find that the outbreak size and duration alone are sufficient in this case to distinguish temporal decline from time-invariant transmission (*p* = 0.014). As use of a single large outbreak can lead to underestimates of immunity, however, we urge caution in interpretation of quantities estimated from this outbreak alone. Further research is needed to distinguish causes of temporal decline in transmission rates.

## Introduction

A large measles outbreak linked to visits to Disneyland theme parks, in which 131 Californians were infected during a period spanning December 2014 to March 2015, made international headlines (Blumberg et al., 2015) and resulted in key changes in vaccine policy in California (SBCS, 2015). This measles outbreak was the largest in California since measles was eliminated from the United States in 2000. The outbreak affected 12 California counties, with 88% of cases occurring in Southern California. Additionally, 16 cases in six other US states, 159 cases in a religious community in Québec, Canada, and one case in Mexico were linked to the California cases. This outbreak, which included almost 30% of the 439 California cases since 2000, provides a unique window into the detailed dynamics of measles epidemiology and transmission in the post-elimination era, particularly outbreaks in large urban settings.

Measles cases have gradually increased overall in the U.S. since the turn of the 21st century (Clemmons et al., 2017), and 2019 has seen the largest number of cases since well before elimination was declared in 2000 (Patel et al., 2019). Investigation is ongoing into whether transmission rates, *i.e.* the number of additional cases that result from a given case on average, are truly increasing and whether the risk of large outbreaks is growing. Ongoing estimation of transmission rates has implications for understanding the dynamics of measles in California and the United States (Gastañaduy et al., 2018a). Previous work concluded that despite the exceptional size of the 2014–2015 outbreak, transmission rates have not necessarily increased (Blumberg et al., 2015).

California local health jurisdictions perform contact investigations during measles outbreaks in order to track and mitigate the spread of measles. These efforts provided detailed information on the timing of cases and their contacts during this large outbreak, as well as information on the ages and measles immunization status of cases. A previous simulation study has argued that not all strategies to prevent the spread of measles during an outbreak are equally effective, due to the challenges inherent in identifying and locating contacts of cases, but that home quarantine for exposed susceptible people may be particularly effective (Liu et al., 2015). Changes in behavior due to news reports during this epidemic and depletion of susceptible people within a home or school may also have led to decreases in transmission over the course of this outbreak. With this analysis, we aim to determine whether there is evidence of a decline in transmission over the course of epidemic.

For the 131 California cases, we use the 37 known epidemiologic linkages in this outbreak, as well as the outbreak duration, to estimate the underlying rate of transmission and its temporal change over the course of the outbreak. Since outbreaks without time-varying trans-mission rates may appear to decline due to the end of the outbreak, a simulation study is used to evaluate the significance of a changing estimate of transmission rates as an estimator of true change in transmission.

## Methods

### Transmission data

Detailed descriptions of this outbreak can be found elsewhere (Zipprich et al., 2015; Adams et al., 2017), and estimates of the reproduction number associated this outbreak have been reported on previously using a subset of the data used for this analysis (Blumberg et al., 2015; Adams et al., 2017). Briefly, the outbreak began in December of 2015 with 42 cases who visited Disneyland theme parks during a 3-day period, and 3 additional cases whose rash onset dates were consistent with exposure during that 3-day period. The case or cases that transmitted to these 45 cases remains unknown. We explore the dynamics of subsequent known transmission, which likely is more typical of transmission in California (Blumberg et al., 2015). An additional 89 California cases were reported over the subsequent 3 months. The outbreak duration was 64 days, where duration of an outbreak is defined to be the amount of time between the rash onset of the first and last cases.

Reported measles cases with travel to Disneyland theme parks, in the greater Los Angeles area, and cases epidemiologically linked to these cases during December 2014 to March 2015 were included in this analysis. To be considered a case, suspected cases were required to meet the Council of State and Territorial Epidemiologists (CSTE) case definition for confirmed measles. The 42 cases who visited Disneyland theme parks during a 3-day period and 3 additional cases whose rash onset dates were consistent with exposure during that 3-day period were all considered index cases for this outbreak. Here, we use “index case” to refer to the progenitor case or cases. Note that this differs from the CDC definition of the index case as the earliest identified case, whereas we allow for multiple index cases who were primary in the transmission chain.

**Fig. 1.**
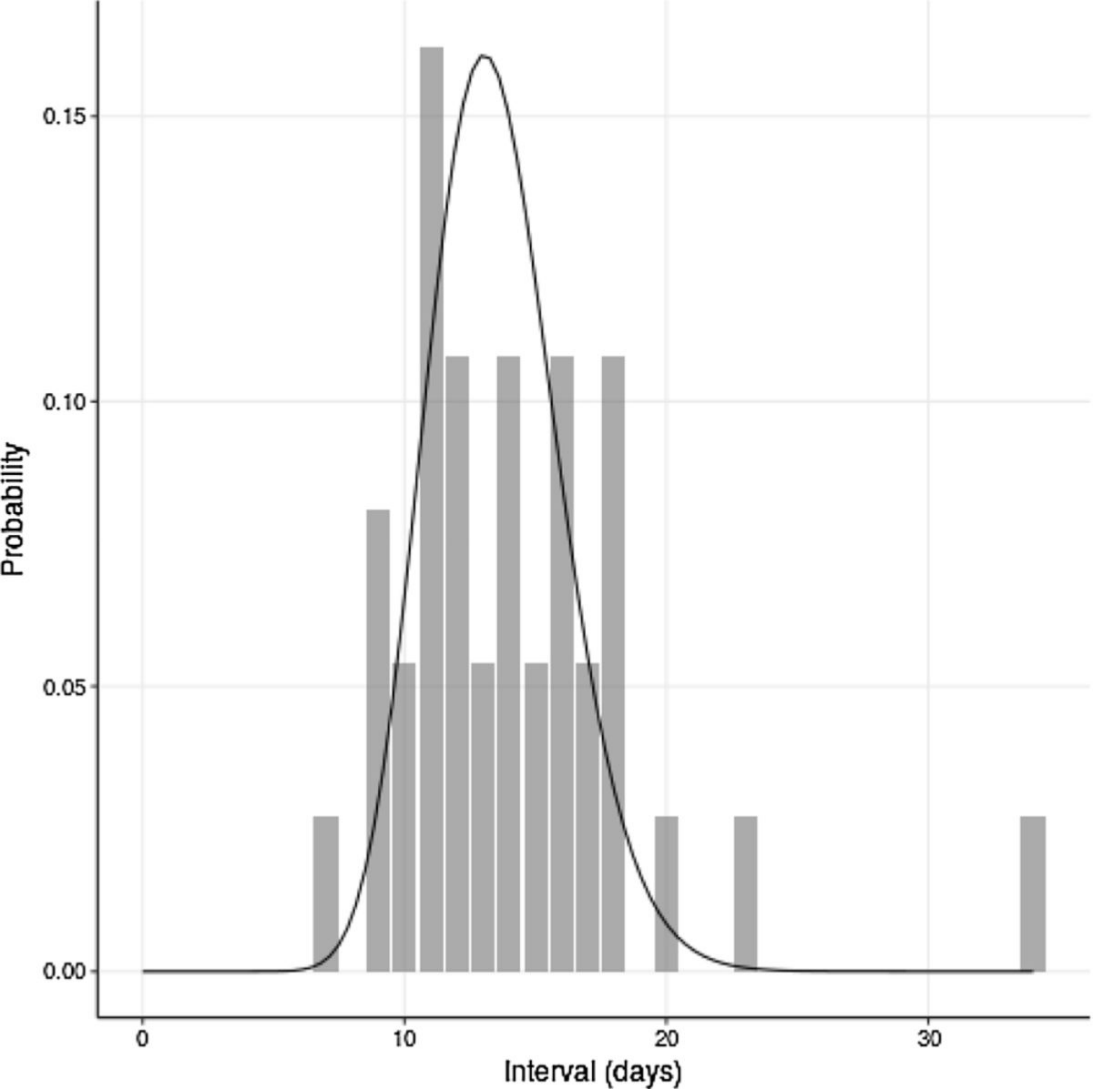
**Serial intervals of reported Disney transmission links** (gray bars) overlaid with estimated serial interval distribution (black curve).

Data analyzed for each case in the outbreak included: rash onset in days from the earliest reported case; vaccination status, including number of measles, mumps, and rubella (MMR) vaccine doses; reporting county; age category; and any known transmission links. The following age categories were used: Young Child (age 0–4 years), School-Age Child (5–18 years) and Adult (19+ years). Epidemiologists in California local health departments and at the California Department of Public Health determined epidemiologically linked cases using the reported contacts of cases. If an individual developed measles symptoms between 8 and 21 days following contact with an infectious case and had no other likely exposure source (within plus or minus 4 days of rash onset), these individuals were assumed to be epidemiologically linked. In one exceptional case, a link with a longer serial interval of 34 days was recorded, in which an immunocompromised individual was infected (Fig. 1). Vaccine status was based on immunization records, where available, and self-report when vaccine records were unavailable.

### Estimation of reproduction numbers

A reproduction number *R* is defined as the number of cases directly infected by a single infective case (Blumberg et al., 2014). The Wallinga-Teunis method of estimation of *R* requires an *a priori* estimate of the probability distribution of the serial interval from the onset of symptoms in a given case *A* to that of a case infected by *A* (Wallinga and Teunis, 2004; Cauchemez et al., 2006; Soetens et al., 2018; Kelly et al., 2018). Given the serial interval distribution, together with the onset time for each case of an outbreak, this method constructs an estimate of likelihood for all possible transmission links between cases. This can then be used to estimate the reproduction number for each case in the outbreak. Letting *w*(*d*) be the probability of an interval of *d* days, this estimation begins by defining a relative likelihood that any given case *j* is the source of infection of any case *i*, by normalizing the likelihoods for all possible sources to total one:

$$p_{\text{ij}}=\frac{w\left(t_{i}-t_{j}\right)}{\sum_{j^{\prime }}w\left(t_{i}-t_{j^{\prime }}\right)}$$

where *t*_*i*_ is the time of symptom onset of case *i*. Index cases are excluded from this estimate by assigning *p*_*ij*_=0 where *i* is an index case. The reproduction number corresponding to each case *j* is then estimated by the number of possible secondary cases weighted by the likelihood of their infection by *j*:

$$R_{j}=\sum_{i}p_{ij}.$$

The relative likelihood *p*_*ij*_ can be adjusted to incorporate prior knowl-edge about transmission chains, by directly defining *p*_*ij*_ = 1 when there is a known link from *j* to *i* and *p*_*ij*_ = 0 when it is known that *j* did not transmit to *i*.

We assumed the serial interval to be gamma distributed with mean of 14.5 days and standard deviation of 3.25 days, discretized to whole numbers of days, consistent with measles transmission intervals ranging from 7 days to 21 days, with mean near 14 days (Gastañaduy et al., 2018b). Wallinga-Teunis estimation was then used to estimate the re-production number *R* for each case in the Disney outbreak, to construct average *R* estimates by two-week periods, and to evaluate the average *R* for vaccinated and unvaccinated individuals and for adults and children.

While the Wallinga-Teunis technique has been used to estimate reproduction numbers (Wallinga and Teunis, 2004; Cauchemez et al., 2006; Soetens et al., 2018; Kelly et al., 2018), it provides an assignment of likelihood to every possible transmission network over all the known cases in the outbreak, under the plausible assumption that the source of infection of one case is independent of the sources of infection of other cases. This likelihood distribution over transmission networks can be used to estimate other quantities of interest, including generation membership.

A probabilistic assignment of generation membership to cases can be estimated as follows. Let *g*_*mi*_ be the likelihood that case *i* belongs to generation *m*. Let *g*_0*i*_ be 1 for all index cases *i* and 0 for all other cases.

For each *m* > 0 in sequence, let

$$g_{\text{mi}}=\sum_{j}p_{\text{ij}}g_{(m-1)j}.$$

We note that when the interval distribution includes very short intervals with positive probability (as the gamma distribution does), this can produce very small but positive likelihoods for very large generation numbers; these can be truncated or combined for simplicity, or the function can be adjusted to remove those implausible small probabilities.

We estimated generation membership for all cases in the Disney outbreak. Likelihoods for generations 5 and up were combined. We then used this assignment to generations to estimate average reproduction numbers by generation.

### Testing for change in R

Multiple studies (Wallinga and Teunis, 2004; Cauchemez et al., 2006; Kelly et al., 2018) have reported estimates of the effective reproduction number (*R*) gradually declining during the course of an outbreak. Can we interpret a declining estimate of *R* as evidence of a true decline of transmission? A transmission process in which contact rates and the probability of effective transmission given a contact are unchanging in time has a fixed expected number of secondary cases per case. This can be described as a process with a fixed “true” *R* in the sense of expected reproduction number, even if isolated outbreaks come and go and the exact number of secondary cases caused by a case – the reproduction number of a given case – varies from case to case within an outbreak. It is of interest to know whether, or how much, realized reproduction numbers of cases, and estimates of their reproduction numbers, may decline temporally in the absence of a true decline in *R*.

Even if estimates of reproduction numbers can be interpreted as declining, without further information it is probably not possible to distinguish between potential causes of decline. Transmission rates can decrease due to quarantine, vaccination, or other control efforts, or due to depletion of susceptibles as individuals are removed from the susceptible pool by becoming infected, or for other reasons such as behavioral changes in susceptible or infective individuals due to concern about an outbreak. We use the term “quenching” of reproduction numbers to refer to a temporal decline in the expected number of secondary cases per case over the course of an outbreak, without reference to the cause of the decline. Thus we do not distinguish depletion of susceptibles from other changes in the outbreak’s dynamics affecting effective transmission rates during its course.

If an estimate of time-changing *R* appears to be quenched, can we conclude that transmission was truly quenched? Can it be used to distinguish quenched transmission from a transmission process in which expected number of secondary cases is in fact constant, but actual number of secondary cases appears quenched? We note that *R* is always zero for the last case of an outbreak. Estimates of average *R* per day tend to generate a smooth curve of values from beginning to end of an outbreak, and while those curves can rise and fall or remain relatively constant for periods during the outbreak, they tend to begin at relatively high values, since early transmission is necessary to the existence of an outbreak, and take on low values at the very end, since the last case of an outbreak necessarily generates no secondary cases. For this reason, a disease process with a time-invariant expected *R*, observed in a series of discrete outbreaks, might yield declining estimates of *R* over time or over generations within each outbreak. Such estimates may decline from a relatively large number to smaller numbers later in the outbreak, perhaps simply as a consequence of the finite duration of each outbreak. Because of these questions, methods are needed to evaluate whether estimates of *R* that decrease with time within an outbreak are evidence of a quenched transmission process.

To this end we used a stochastic model of measles transmission to examine the estimates obtained for changing and unchanging true *R* values, and tested the null hypothesis of unchanging *R* against the estimates seen for the Disney outbreak. We simulated outbreaks using a standard Galton-Watson branching process with a negative binomial distribution of secondary cases per primary case, parameterized by the mean number of secondary cases, *R*, and a dispersion parameter *k* (Blumberg and Lloyd-Smith, 2013a; Lloyd-Smith et al., 2005). Except where otherwise noted, simulations were conducted using a dispersion parameter *k* = 0.40, previously estimated from measles outbreaks in California during the post-elimination era (2000–2015) (Ackley et al., 2017). Sensitivity of simulation results to the value of *k* was examined by using a range of *k* values for estimates of quenching (see below).

Outbreaks were simulated with 45 index cases, as in the Disney outbreak. Given an outbreak generated by this branching process, we assigned onset dates to all cases using the interval distribution described above. We assigned onset dates to the 45 index cases by assuming a unobserved “pre-index case” to be the source of transmission for all the index cases. Each index case was assigned an onset date by adding an interval drawn from the interval distribution to the onset date of its source case. Onset dates were then shifted to let the earliest onset date of the 45 index cases be labeled day 0. Simulated outbreaks generated in this way were compared to the Disney outbreak, both directly in their size (number of cases) and duration (onset date of last case), and using statistics estimated by the Wallinga-Teunis formulas.

### Quenching in Wallinga-Teunis estimates as an estimator of true quenching

To test whether the above method of estimation of *R* by generation can detect true decline, or quenching, in *R*, we applied the estimation procedure to simulated outbreaks in which *R* was assumed constant across generations. We estimated *R* by generation using 1024 simulated outbreaks with each of a range of true values of (constant) *R* and dispersion parameter *k*. Simulated outbreaks were conditioned on size of 131, the size of the Disney outbreak in California. 37 known trans-mission links, equal to the number reported for the Disney outbreak, were assigned at random. The methods developed above were then used to estimate *R* as a function of generation. Because of the above mentioned tail of improbably large generation numbers, generation length of the outbreak was estimated as 1/14 the number of days from first to last onset date, rounded down to the nearest integer, plus one, and only generations up to that length were estimated. This sequence of *R* values was fit to an exponentially decaying curve, *R* = *R*_*i*_*e*^−*τm*^, for each generation *m*, by nonlinear least-squares fitting, using the nlmrt package in R. The same estimation procedure was applied to the Disney outbreak data. The method of moments estimate for unquenched *R*, *R* = (131 − 45)/131, (Blumberg and Lloyd-Smith, 2013b) with *τ* = 0, was used to generate a sample of estimates $\hat{\tau }$ from simulation. The $\hat{\tau }$ estimated for the Disney case was tested against this sample to evaluate the null hypothesis that the Disney outbreak was generated by a disease process with an unchanging secondary case distribution. Specifically, we observed the fraction of simulations for which the estimated $\hat{\tau }$ exceeded the observed value, and doubled it to obtain a two-sided test.

We additionally collected estimates of $\hat{\tau }$ across a range of secondary case distribution parameters, *R* =0.5, (131 − 45)/131, and 0.8, and *k* = 0.1, 0.4, 1, and 1,000,000 (the latter an approximation of the limit *k*→ ∞, which produces a Poisson distribution), to test the likelihood of estimating $\hat{\tau }$ values distinct from zero, using a Bonferroni-corrected *α* of 0.05/12 to account for the 12 parameter combinations tested.

### Size and duration as an indicator of quenching

Following the above test for quenching in estimated *R* values by generation, we tested whether size *and duration* (in time, not generation) of the outbreak alone can distinguish quenched from unquenched transmission.

We evaluated the ability of an unquenched transmission process to generate outbreak size and duration pairs similar to those of the Disney outbreak by using the frequency of size within 5 cases and durations within 5 days of the target values in simulated transmission chains as an estimate of likelihood. Simulations were generated using three constant *R* values: the maximum likelihood *R* given outbreak size alone, which is the classical estimate *R* = (131 − 45)/131; (Blumberg and Lloyd-Smith, 2013b) the maximum likelihood value of *R* given size and duration, estimated by sampling likelihood across the range 0.5 ≤ *R* ≤ 2.5; and *R* fit to the Disney outbreak’s duration using Robbins-Monro estimation (Robbins and Monro, 1951).

We then estimated a maximum likelihood fit of exponentially quenched *R* = *R*_*i*_*e*^−*τm*^ per generation *m*, by sampling from a range of *R*_*i*_ and *τ* values as above, including the unquenched case *τ* = 0. We compared the joint distributions of size and duration generated by time-invariant *R* to those generated by exponentially quenched *R* and by the sequence of *R* values estimated by the Wallinga-Teunis method from the Disney outbreak, with the dispersion parameter *k* = 0.40 previously estimated for measles in California.

We used kernel density fitting to approximate the likelihood function estimated by sampling quenched and unquenched (*R*, *τ*) pairs, and used the likelihood ratio test with one degree of freedom to evaluate the null hypothesis that an unquenched secondary case distribution de-scribes the Disney outbreak.

## Results

### Transmission data

**Fig. 2.**
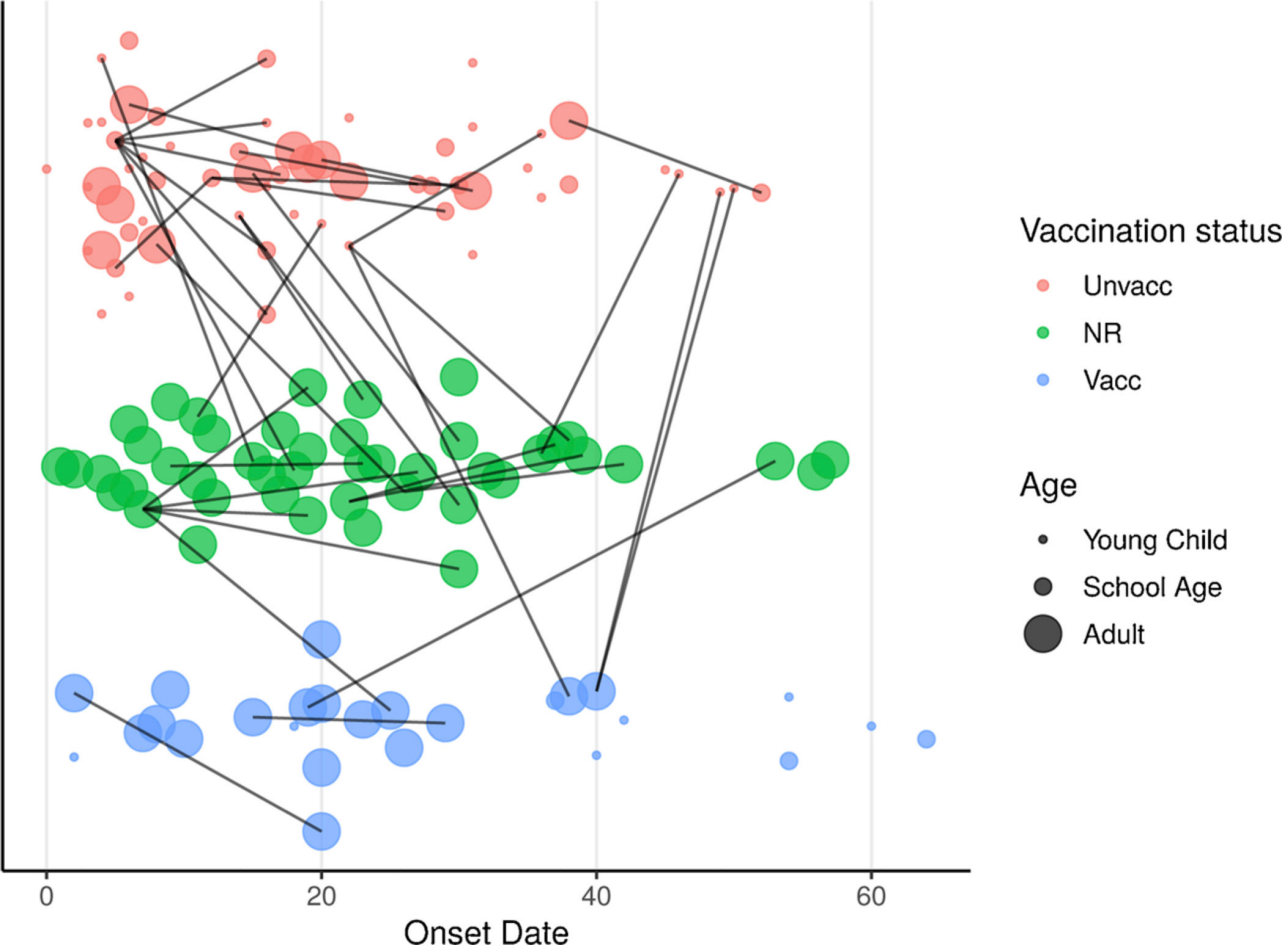
**Transmission network of 2014–2015 outbreak** indicating onset date, age, and vaccination status for each case and known transmission links.

We report the time course of the epidemic and transmission links with vaccine status and age (Fig. 2).

The serial interval distribution implied by reported transmission links in the Disney outbreak is compared to the theoretical interval in Fig. 1.

### Estimation of reproduction numbers

**Fig. 3.**
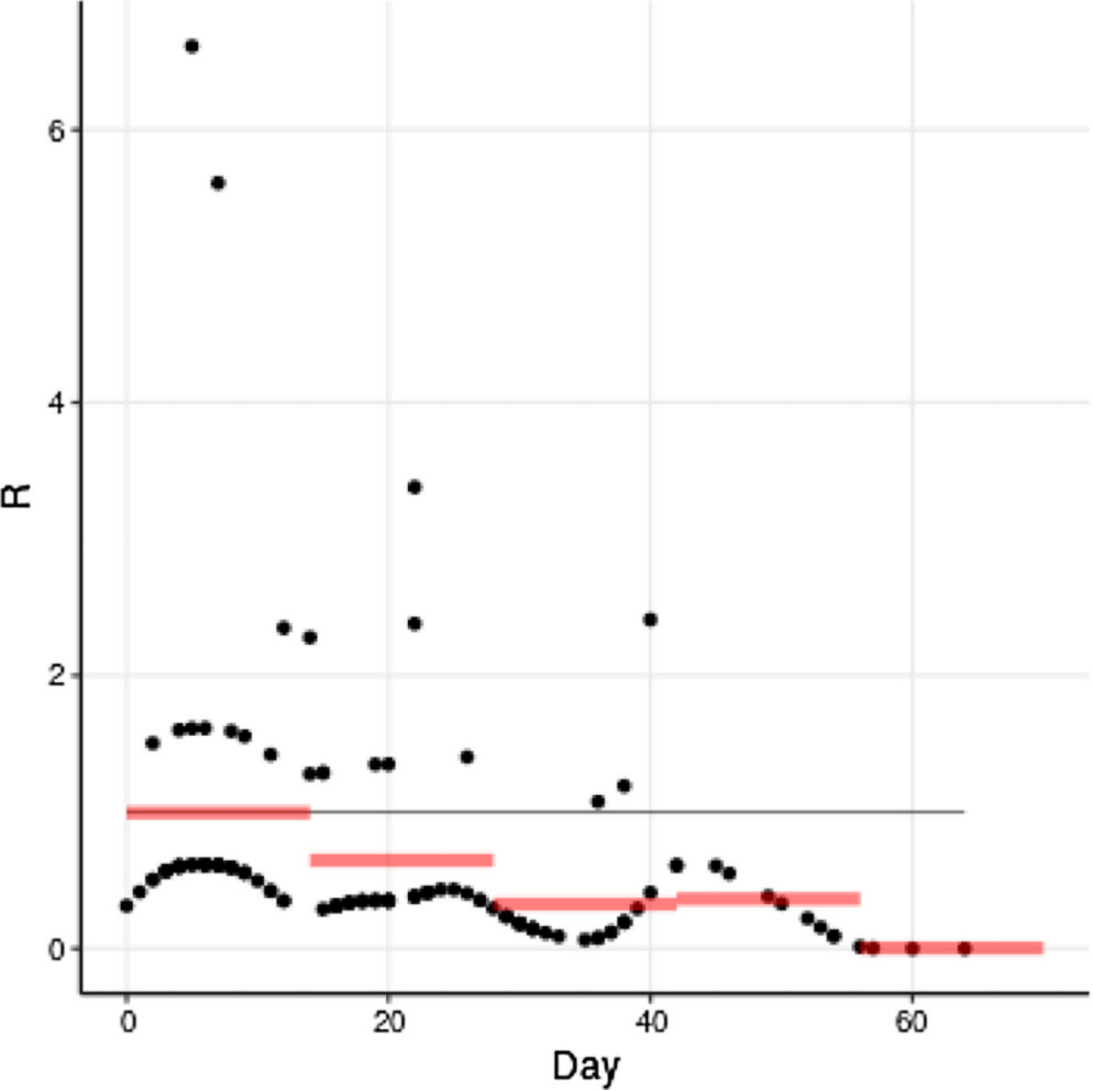
**Estimated reproduction number** *R* **for each case by date and aver-aged over two-week periods**, taking into account reported index cases and transmission links. Cases

Estimated reproduction numbers *R* per case, while widely variable at times due to variability in number of reported secondary cases, exhibit an overall downward trend, while two-week averages of *R* show a definite downward trend (Fig. 3).

**Fig. 4.**
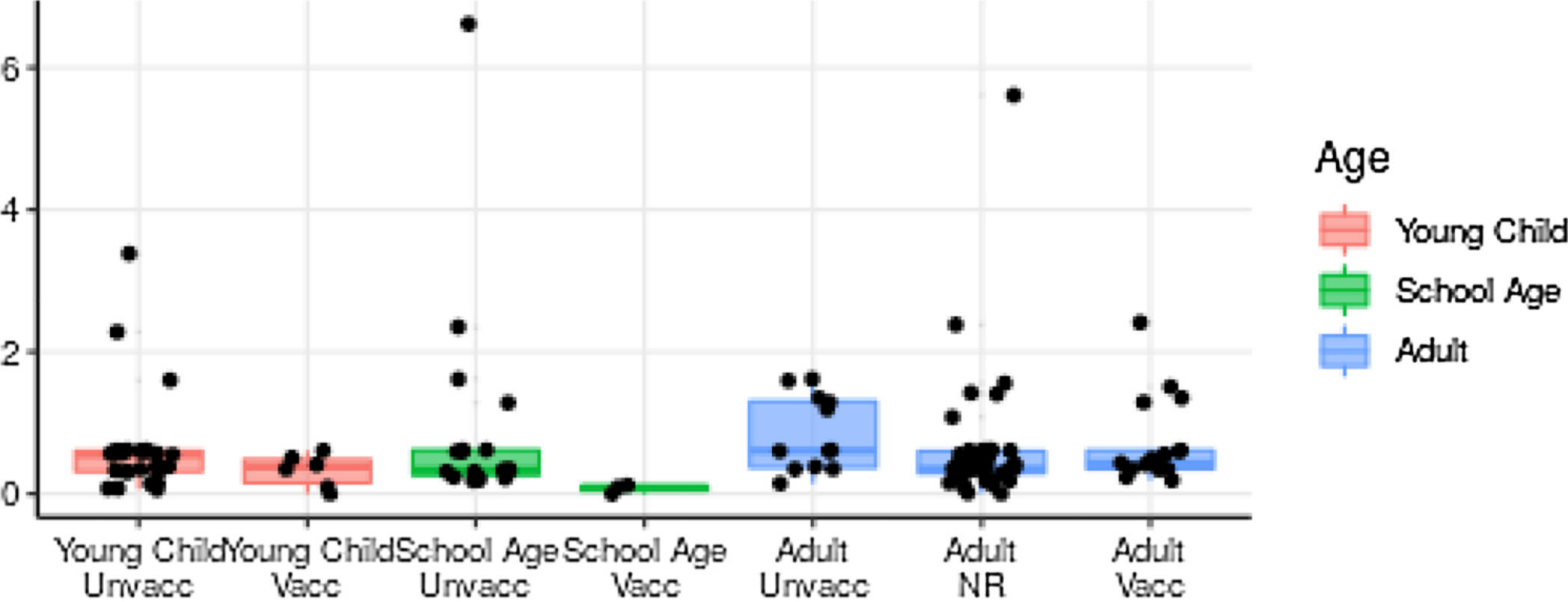
**Estimated number** *R* **for each case, by age and vaccination status**. Estimates of *R* by case are shown in black, and box-and-whisker plot indicates median and quartiles.

When *R* estimates are stratified by vaccine status and age, transmission from vaccinated children of school age may be lower than average while transmission from unvaccinated adults may be higher, though inference is limited by the small sample size (Fig. 4). known to have transmitted have higher estimated *R* than others because the number of known links is added to the expected number of unknown links.

**Fig. 5.**
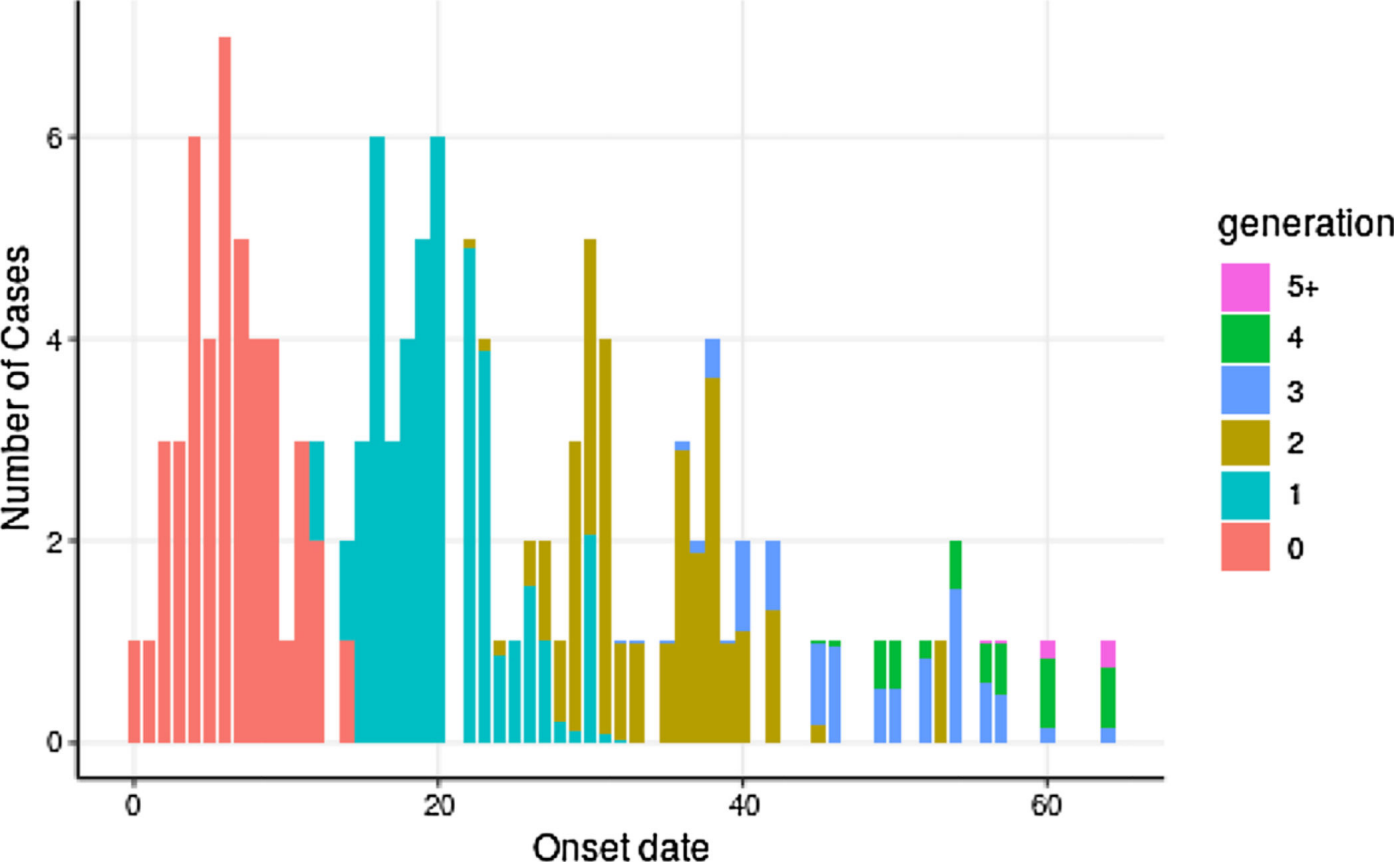
**Estimated generation membership** of cases by day. Generation membership is estimated using the Wallinga-Teunis method, including information about known transmission links (see text for details). At each day, the estimated number of cases belonging to each generation is shown as a colored bar.

**Fig. 6.**
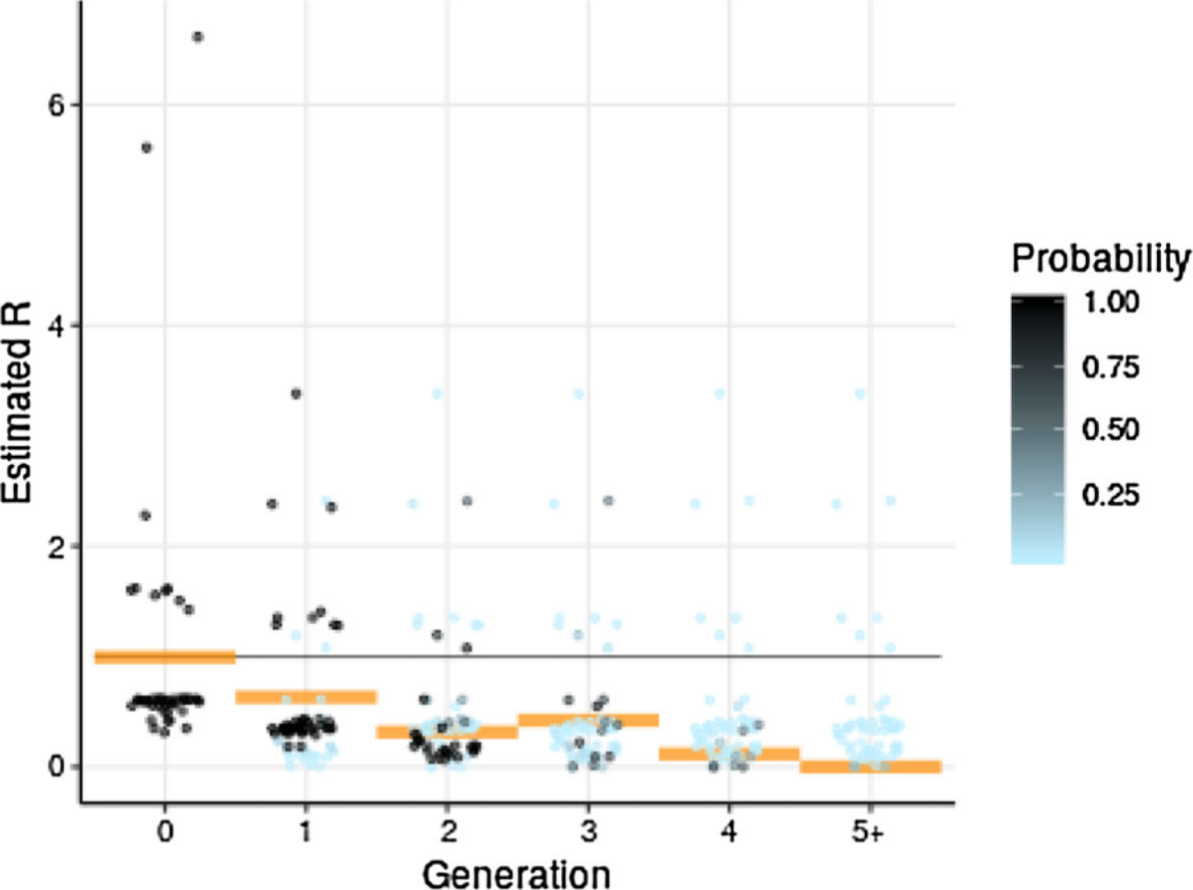
**Estimated reproduction number** *R* **by generation and estimated probability of generation membership per case**. Mean estimated *R* by generation is plotted as orange bars, and probability of membership of each case in each generation is plotted as dots shaded from light blue to black. Points of probability zero are omitted. Note that there are relatively few points of probability near ½. Note also the similarity between estimated *R* by generation and estimated *R* by two-week period (Fig. 3). Because of this similarity, this paper focuses on estimation of *R* by generation, while estimation of *R* by date is a potential subject of future research. (Fig. 7).

For each case, we obtained an estimate of the probability that the given case appeared in each particular generation. Summing these probabilities by day yields the expected number of cases in each generation over time. These estimates (Fig. 5) indicate that the outbreak likely spanned 4 to 5 generations of transmission, and that generation approximately coincides with apparent waves of cases. Combining generation estimates with the above *R* estimates to produce an estimate of average *R* per generation yields a descending sequence closely comparable to the earlier estimate of *R* by two-week interval (Fig. 6).

### Testing for change in R

#### Quenching in Wallinga-Teunis estimates as an estimator of true quenching

The Disney outbreak was tested against a null hypothesis of unquenched transmission by fitting an exponentially declining reproduction number by generation to estimated *R* values per case, and comparing to simulated outbreaks with unchanging true *R*. A similar process of Wallinga-Teunis estimation and fitting of a quenched curve has been used in comparison and forecasting of Ebola virus disease (EVD) case counts (Worden et al., 2019).

We simulated stationary branching process chains (with constant *R*) of size equal to the Disney outbreak, varying the value of *R* and of the dispersion parameter *k*. Based on these simulations, we then used the Wallinga-Teunis method to estimate *R* per case (as for the actual data). Then, we also estimated generation membership in the same way, allowing us to estimate *R* by generation. Finally, we fit an exponentially decaying curve *R* = *R*_*i*_*e*^−*τm*^ per generation *m* to that sequence, by fitting values of the generation 0 reproduction number *R*_*i*_ and quenching rate *τ*. This yielded estimates of the quenching rate that were significantly distinct from zero in all scenarios at a Bonferroni-corrected *α* of 0.05/12

The quenching rate $\hat{\tau }$ estimated for the 131-member Disney out-break, compared to the distribution of quenching rates estimated for simulated outbreaks conditioned on size 131, with *R* = 0.656 (= (131 − 45)/131), *k* = 0.40, and *τ* = 0, was significantly different from zero with *p* = 0.012.

### Size and duration as an indicator of quenching

**Fig. 8.**
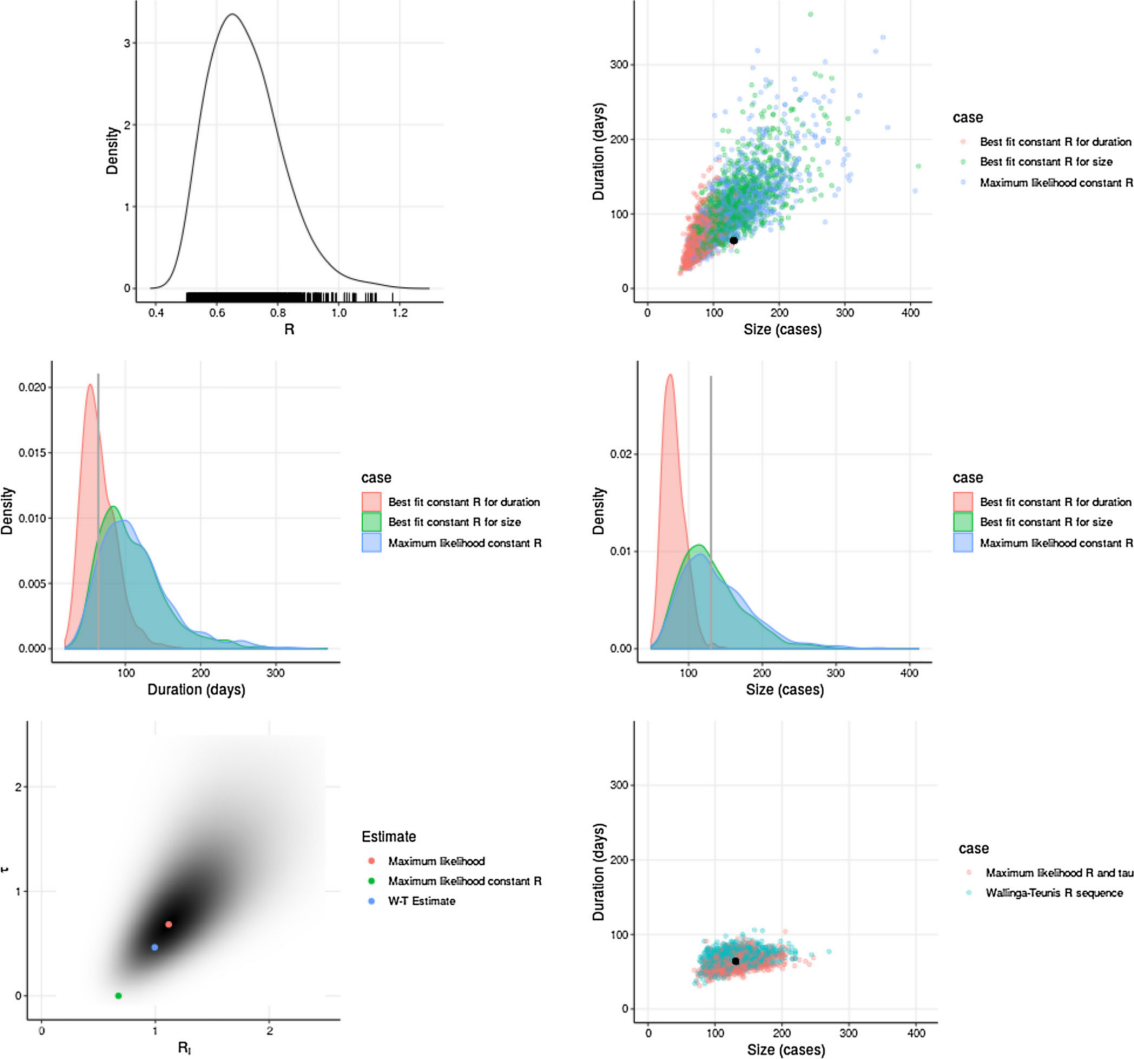
**Size and duration in branching processes** with constant *R* and with quenched *R*, compared to Disney outbreak. **(Top left)** Likelihood of outbreak size within 5 cases and duration within 5 days of Disney measurements in simulation with constant *R*; **(Top right)** Distribution of size and duration given maximum likelihood constant *R*, constant *R* fit to Disney outbreak duration only, and constant *R* fit to Disney outbreak size only (black dot is size and duration of Disney outbreak); **(Middle left)** Distribution of outbreak duration given three constant *R* estimates (vertical line is duration of Disney outbreak); **(Middle right)** Distribution of outbreak size given three constant *R* estimates (vertical line is size of Disney outbreak); **(Bottom left)** Likelihood surface for *R*_*i*_ and *τ* parameters describing quenched and unquenched *R*; **(Bottom right)** Distribution of size and duration given maximum likelihood quenched *R* and quenched *R* sequence estimated by Wallinga-Teunis method (black dot is size and duration of Disney outbreak). Simulated with *k* = 0.40.

We found that using the serial interval distribution discussed above, the size and duration of the Disney outbreak are rarely observed jointly in branching processes with any constant value of *R* (Fig. 8).

A likelihood ratio test comparing the likelihood of this size and duration under the maximum likelihood quenched *R* model to the maximum likelihood constant *R* model (*χ*^2^ distribution with one degree of freedom) found that quenching rate *τ* was significantly different from 0 (*p* = 0.014).

## Discussion

We find evidence of changing transmission conditions over the course of a large measles outbreak linked to Disneyland theme parks. According to our estimates of the reproduction number *R* over time, *R* started out close to one and then fell over the course of the outbreak by a factor of approximately two-thirds with each subsequent generation. However, our simulation studies indicate that these estimates of quenching in *R* are significantly biased away from zero for a process with no quenching, and thus may overestimate both the true quenching rate and initial reproduction number. Nonetheless, the observed rate of decline in transmission is significantly in excess of the decline in transmission typically observed during comparably sized outbreaks with an unchanging transmission process.

Our study has several strengths. First, we used detailed data from the California Department of Public Health that was collected during the contact investigations performed during this outbreak. These data provide an opportunity to describe transmission and quantify changes in transmission conditions during a large, predominantly urban out-break in the postelimination era. Second, we rejected the null hypothesis of no quenching during this outbreak using two different methods: showing that the observed quenching exceeded the expected measured quenching using Wallinga-Teunis estimation, and showing from simu-lation that the outbreak’s duration was atypically short for an outbreak with no quenching.

The Wallinga-Teunis method and derived techniques have been used to infer changes in *R* for outbreaks of measles (Chong et al., 2018; Linton, 2018) and other diseases (Wallinga and Teunis, 2004; Cauchemez et al., 2006; Goldstein et al., 2009; Soetens et al., 2018; Kelly et al., 2018). Our results suggest that such observed changes may in some cases be artifactual, since a finite sized outbreak will by definition have no transmission in the final generation and may proceed from high levels to lower levels of transmission in a roughly continuous fashion. That is, observed quenching conditioned on occurrence of a sizeable outbreak overestimates changes in transmission conditions over time.

Our study has several limitations. First, we observed a single, large outbreak that may not be typical of measles transmission in California, or the United States. These results should be validated against a more complete set of outbreaks. Second, while we found evidence for quenching, we cannot distinguish competing causes of changing transmissibility over time. For example, it is plausible that media attention and public health response played a large role in quenching during this outbreak, but during smaller outbreaks this quenching effect could be more minimal or non-existent. A study that determined how transmission over the course of an outbreak varied with outbreak size might help to falsify or fail to falsify specific explanations of quenching. For example, if quenching rates are no larger in outbreaks that received significant amounts of media attention, that would lend credibility to the hypothesis that saturation of local micronetworks plays a larger role in the quenching process than behavioral change due to information. Further research will have to examine possible sources of quenching. Third, the serial interval we used, while similar to previous interval distributions and based on empirical estimates, may not correspond precisely to the transmission conditions during this outbreak. Wallinga-Teunis estimation can be biased if the serial interval is estimated from a data set in which control measures were used, since intervals can be shorter before the start of control and longer after (Lipsitch and Bergstrom, 2004). We note that the observed serial intervals for the known epidemiologic linkages closely match the serial interval distribution we chose for the main analysis (Fig. 1). Since it is possible this is a biased set of epidemiologic linkages, we also performed a sensitivity analysis with a slightly larger serial interval (mean 14.5 days, s.d. 3.25 days) and found that our conclusions were unaltered. We tested secondly a smaller serial interval estimated from historical data (Klinkenberg and Nishiura, 2011) (mean 11.2 days, s.d. 2.62 days) and found that the outcomes were qualitatively the same, though *p*-values did not fall below the significance threshold in this case. We note that the latter serial interval distribution is shorter than seen in the 2014–2015 outbreak.

We note that selecting on the largest California outbreak since elimination may tend to lead to overestimates of both the average reproduction number, *R* (Blumberg et al., 2015), and the initial reproduction number, *R*_*i*_, presented here. While the inclusion of cases outside California linked to this outbreak would likely yield a longer time series of *R* values and somewhat higher values for the cases included, the outbreak should be considered an instance of unusually sustained transmission regardless. The average reproduction number or reproduction numbers over time should not be inferred from a single, highly unusual outbreak.

In conclusion, we observed evidence of quenching of transmission during this outbreak in excess of what would be expected from large outbreaks with no true decline in transmission rates. Further research is required to determine the source of the changes in transmission conditions over the course of this outbreak and the true magnitude of the change.

## Figures and Tables

**Fig. 7.**
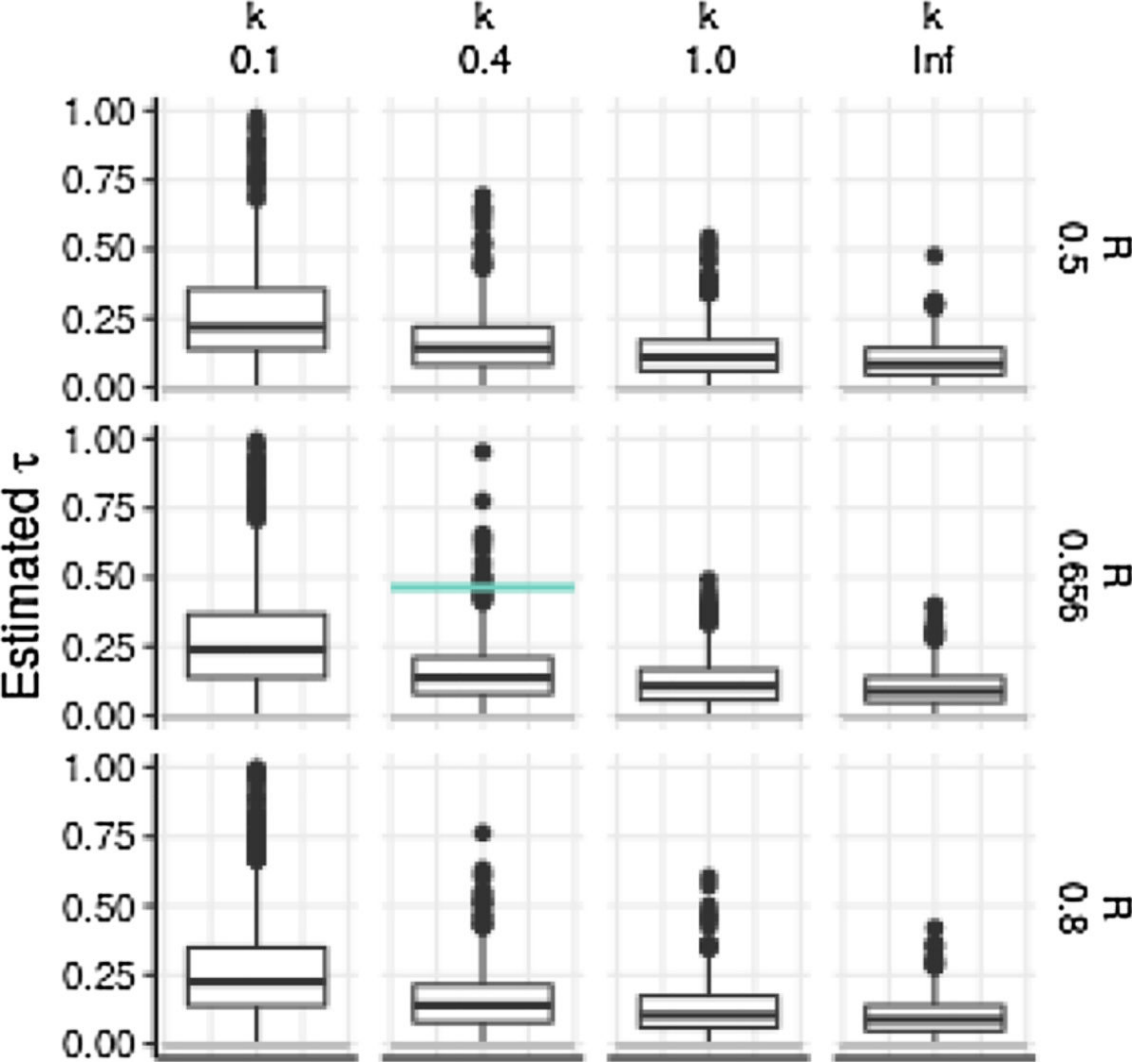
**Distribution of estimates** of rate of decline of *R* by generation in simulations with constant *R*, against true *R* (top to bottom) and *k* (left to right). Rates of quenching of transmission $\hat{\tau }$ obtained by fitting function *R* = *R*_*i*_*e*^−*τm*^ to Wallinga-Teunis estimated *R* by generation *m* in simulated outbreaks, conditioned on outbreak size of 131 cases. Horizontal gray line shows the true value *τ* = 0, and horizontal colored line shows quenching rate $\hat{\tau }$ estimated for Disney outbreak, illustrating the significance test reported in the text. Boxes mark median and interquartile range of estimates. The *k* = 0.1 case includes outliers $\hat{\tau }$ not shown.
